# Marker-assisted introgression of a QTL region to improve rust resistance in three elite and popular varieties of peanut (*Arachis hypogaea* L.)

**DOI:** 10.1007/s00122-014-2338-3

**Published:** 2014-06-14

**Authors:** Rajeev K. Varshney, Manish K. Pandey, Pasupuleti Janila, Shyam N. Nigam, Harikishan Sudini, M. V. C. Gowda, Manda Sriswathi, T. Radhakrishnan, Surendra S. Manohar, Patne Nagesh

**Affiliations:** 1 International Crops Research Institute for the Semi-Arid Tropics (ICRISAT), Hyderabad, 502324 India; 2 CGIAR Generation Challenge Programme (GCP), c/o CIMMYT, 06600 Mexico, DF Mexico; 3 University of Agricultural Sciences (UAS), Dharwad, 580005 India; 4 Directorate of Groundnut Research (DGR), Junagadh, 362001 India

## Abstract

*****Key message***:**

**Successful introgression of a major QTL for rust resistance, through marker-assisted backcrossing, in three popular Indian peanut cultivars generated several promising introgression lines with enhanced rust resistance and higher yield.**

**Abstract:**

Leaf rust, caused by *Puccinia arachidis* Speg, is one of the major devastating diseases in peanut (*Arachis hypogaea* L.). One QTL region on linkage group AhXV explaining upto 82.62 % phenotypic variation for rust resistance was validated and introgressed from cultivar ‘GPBD 4’ into three rust susceptible varieties (‘ICGV 91114’, ‘JL 24’ and ‘TAG 24’) through marker-assisted backcrossing (MABC). The MABC approach employed a total of four markers including one dominant (IPAHM103) and three co-dominant (GM2079, GM1536, GM2301) markers present in the QTL region. After 2–3 backcrosses and selfing, 200 introgression lines (ILs) were developed from all the three crosses. Field evaluation identified 81 ILs with improved rust resistance. Those ILs had significantly increased pod yields (56–96 %) in infested environments compared to the susceptible parents. Screening of selected 43 promising ILs with 13 markers present on linkage group AhXV showed introgression of the target QTL region from the resistant parent in 11 ILs. Multi-location field evaluation of these ILs should lead to the release of improved varieties. The linked markers may be used in improving rust resistance in peanut breeding programmes.

**Electronic supplementary material:**

The online version of this article (doi:10.1007/s00122-014-2338-3) contains supplementary material, which is available to authorized users.

## Introduction

Peanut or groundnut (*Arachis hypogaea* L.) is one of the most important oilseed and food crops having a large impact on the livelihoods of poor farmers in the semi-arid tropics (SAT). It is cultivated on over 24 million hectares (M ha) with a global production of about 38 million tonnes (Mt) (FAOSTAT 2012). Several biotic and abiotic constraints limit the realization of the full genetic potential of modern improved peanut varieties. For instance, rust disease caused by *Puccinia arachidis* Speg. is one of the widespread diseases that severely affects peanut productivity in tropical countries. Many other species of this fungal pathogen are known to damage a majority of grain and forage legumes worldwide (Sillero et al. 2006).

Several popular peanut varieties have been phased out of farmers’ fields in the recent past due to heavy yield losses caused by their susceptibility to foliar fungal diseases. In general, disease control is possible with fungicides (4–8 sprays based on disease severity) but a majority of farmers in the SAT cannot afford them since they lack the resources and technical expertise required to use them effectively (Subrahmanyam et al. 1984). Moreover, the use of fungicides is neither a cost-effective approach nor a healthy practice for the environment and human health. Under these circumstances, a genetic approach involving introgression of disease resistance into modern and popular cultivars seems to be ideal. Conventional breeding has been successful in introgressing resistance in peanut breeding programmes. However, it is labour intensive and time consuming. Recent advances in crop genomics facilitate the identification of molecular markers associated with target trait(s) that can be deployed to select a superior line in a breeding programme. This process, known as ‘genomics-assisted breeding’ (Varshney et al. 2005), has been used to improve several traits in some legume crops (Varshney et al. 2006, 2010).

Two recombinant inbred line (RIL) mapping populations, namely ‘TAG 24’ (susceptible) × ‘GPBD 4’ (resistant) and ‘TG 26’ (susceptible) × ‘GPBD 4’ were used earlier to map rust resistance (Khedikar et al. 2010; Sujay et al. 2012). Initially, a partial genetic map comprising of 56 marker loci was developed on the ‘TAG 24’ × ‘GPBD 4’ RIL population and a major quantitative trait loci (QTL) for rust resistance explaining 55.20 % phenotypic variation (PV) was identified (Khedikar et al. 2010). The nearest marker to the QTL, IPAHM103 (developed by Cuc et al. 2008), was found to be tightly linked with rust resistance. Subsequently, saturation of genetic maps with additional 132 SSR marker loci and comprehensive QTL analyses provided not only additional linked co-dominant markers (GM2009, GM1536, GM2301 and GM2079) but also increased the resolution of QTL with a more accurate estimation of QTL effect (82.96 % PV) (Sujay et al. 2012). The reliability of these linked markers was confirmed by validating them on a set of resistant and susceptible genotypes (Khedikar et al. 2010, unpublished data).

This study was undertaken to introgress the QTL region controlling rust resistance into two elite peanut varieties (‘TAG 24’ and ‘ICGV 91114’) and one old but popular variety (‘JL 24’) through marker-assisted backcrossing (MABC). Four linked markers (IPAHM103, GM1536, GM2301 and GM2079) from the QTL region were used to select the lines to make backcrosses for the next generation in MABC. After making 2–3 backcrosses and selfing the backcross progenies, several backcross-derived introgression lines (ILs) with enhanced rust resistance and better yield compared to the respective recurrent parent genotype were developed.

## Materials and methods

### Plant material

Three rust-susceptible Indian peanut varieties, namely ‘ICG 91114’, ‘JL 24’ and ‘TAG 24’, all Spanish Bunch types (*A. hypogaea* subsp. *fastigiata* var. *vulgaris*), were selected for introgression of rust resistance. ‘ICGV 91114’ is a widely adapted, high-yielding, drought-tolerant and early-maturing (100–105 days) variety. It is an ICRISAT-bred variety which was identified through farmer participatory varietal selection in Anantapur district of Andhra Pradesh (India), and released for cultivation in 2006. It was selected from the cross ‘ICGV 86055’ × ‘ICGV 86533’ following bulk-pedigree method.

‘JL 24’, is a high-yielding, drought-tolerant and early-maturing (100–110 days) variety selected from ‘EC 94943’, an introduction from Taiwan, at the Oilseeds Research Station, Jalgaon, Maharashtra (India). It was released for cultivation in India during 1979 and has been very popular in India and elsewhere.

‘TAG 24’, a popular variety, is a high-yielding and early-maturing (100–110 days) variety with high harvest index is used extensively for confectionery purpose. This variety, a derivative of the cross ‘TGS2’ × ‘TGE1’, was bred at Bhabha Atomic Research Center, Trombay (India) and released for cultivation in 1992. All three varieties are susceptible to rust and late leaf spot (LLS) caused by *Phaeoisariopsis personata* (Berk. and M.A. Curtis) Van Arx.

‘GPBD 4’ is a highly resistant variety to rust and LLS and was selected as a donor parent in the MABC programme as it was used to identify the QTL region for rust resistance. It was derived from the cross ‘KRG 1’ × ‘CS 16’ (‘ICGV 86855’) and is a second cycle derivative of inter-specific hybridization with a desirable combination of mid-early maturity, high yield, high pod growth rate and pod and kernel with high oil content (Gowda et al. 2002).

### Molecular markers

A total of four linked markers, namely IPAHM103, GM1536, GM2301 and GM2079, were used to select lines carrying the ‘GPBD 4’ allele for rust resistance to make backcross progenies possessing the QTL genomic region from the resistant donor genotype ‘GPBD 4’ (Table 1). To estimate the recovery of the recurrent parent genome, a total of 13 SSR markers—GM2009, GM1536, GM2301, GM2709, IPAHM103, GM1954, TC4G02, Seq2B10, GM2069, GM2053, GM1996, GM1883 and GM1502 from the carrier linkage group AhXV were used for analyzing the select ILs.

**Table 1:** Sequence and amplification information for linked markers for rust resistance in peanut

Linked markers	Marker type	Sequence	Annealing temp	Resistance parent allele (bp)	Susceptible parent allele (bp)
IPAHM103	Dominant	Forward: GCATTCACCACCATAGTCCA	60.0	154	130
		Reverse: TCCTCTGACTTTCCTCCATCA			
GM1536	Co-dominant	Forward: AAAGCCCTGAAAAGAAAGCAG	60.3	473	482
		Reverse: TATGCATTTGCAGGTTCTGGT			
GM2301	Co-dominant	Forward: GTAACCACAGCTGGCATGAAC	60.3	127	136
		Reverse: TCTTCAAGAACCCACCAACAC			
GM2079	Co-dominant	Forward: GGCCAAGGAGAAGAAGAAAGA	60.0	416	436
		Reverse: GAAGGAGTAGTGGTGCTGCTG			

### DNA extraction, PCR and marker genotyping

DNA was extracted from fresh leaves of the parental genotypes, F_1_s and backcross (BC) progenies using 25-day-old seedlings by means of the modified cetyltrimethyl ammonium bromide (CTAB) extraction method (Cuc et al. 2008). DNA quality and quantity were checked on 0.8 % agarose gels and DNA concentration was normalized to ~5 ng/μl for further genotyping with linked markers (Table 1).

Linked SSR markers were used for amplification using polymerase chain reaction (PCR) following conditions mentioned in Khedikar et al. (2010) and Sujay et al. (2012). PCR reactions were prepared by mixing ~5 ng of genomic DNA, 2 pmol of each primer, 2 mM of dNTPs, 2 mM MgCl_2_, 1X amplification buffer and 0.1 U of *Taq* DNA polymerase (Qiagen, Hilden, Germany). PCR for these SSR markers were performed in a 5 µl volume following a touchdown profile in an ABI thermal cycler (Applied Biosystems, Foster City, CA, USA). The touchdown PCR amplification profile consisted of an initial denaturation (3 min at 94 °C) followed by an initial five cycles (94 °C for 20 s (s), 65 °C for 20 s and 72 °C for 30 s) with a 1 °C decrease in temperature in each cycle. This was followed by 35 cycles (94 °C for 20 s with constant annealing temperature of 59 °C for 20 s and 72 °C for 30 s) and lastly, an extension for 20 min at 72 °C. The products were tested on 1.2 % agarose gels to check the amplification. The PCR amplicons of the linked markers were separated using polyacrylamide gel electrophoresis (PAGE) (Tegelstrom 1992).

### Backcrossing

**Fig. 1:**
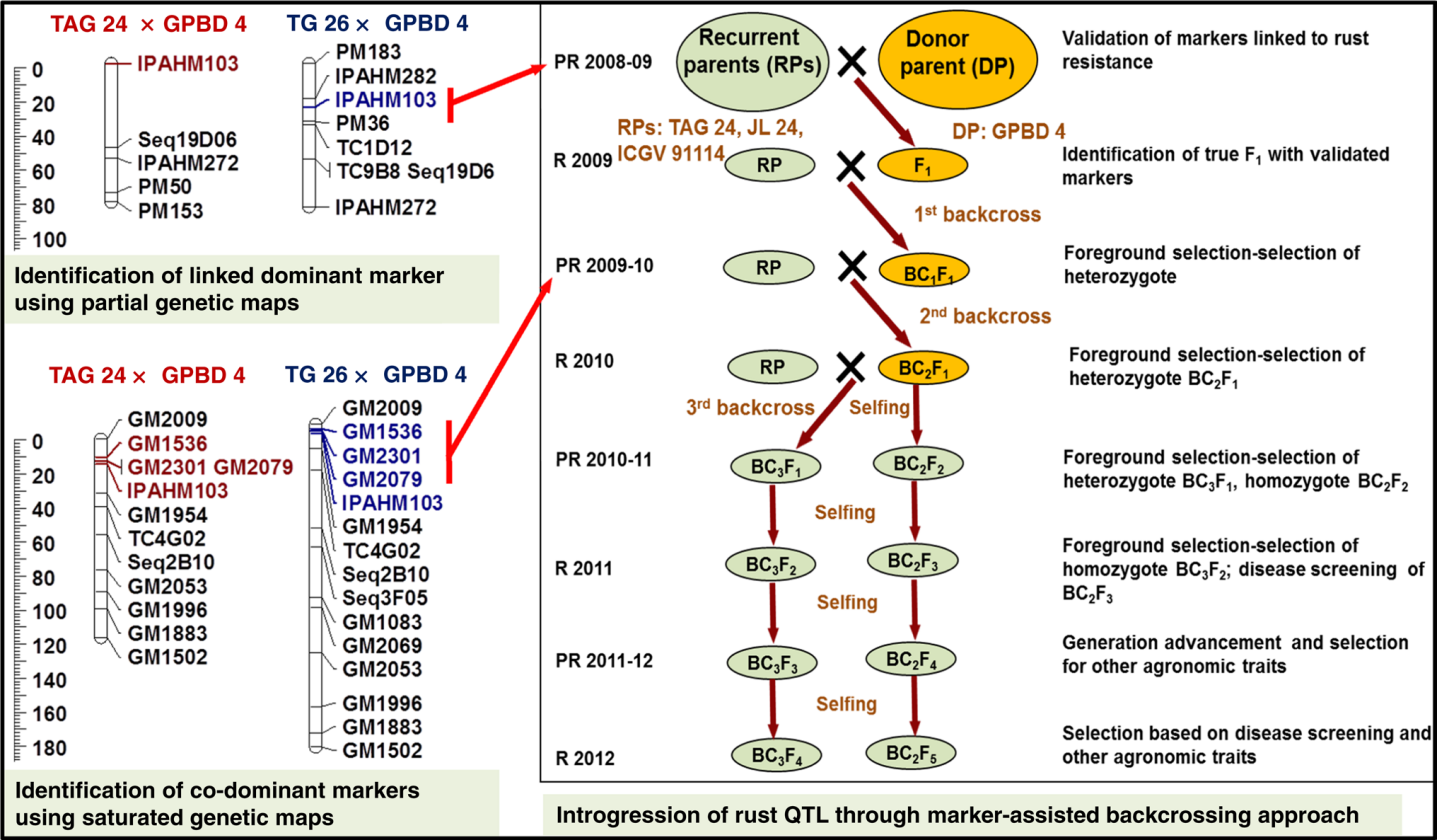
An overview on improvement of rust resistance in peanut through marker-assisted backcrossing (MABC). This illustration shows (**a**) the linkage groups of partial genetic maps from two mapping populations (‘TAG 24’ × ‘GPBD 4’ and ‘TG 26’ × ‘GPBD 4’) where dominant marker IPAHM103 was found linked with rust resistance. This marker after validation was used in early MABC generations, **b** linkage maps after saturation where three additional linked co-dominant markers (GM1536, GM2301 and GM2079) were mapped in the same region and been deployed in MABC programme from BC_1_F_1_ generation onwards, and **c** displays schematic presentation of MABC approach for introgressing rust resistance QTL in three elite (‘TAG 24’, ‘JL 24’ and ‘ICGV 91114’) cultivars using the same donor (‘GPBD 4’) which was used in QTL mapping

All three target cultivars were used as female parents to make independent crosses (‘ICGV 91114’ × ‘GPBD 4’, ‘JL 24’ × ‘GPBD 4’ and ‘TAG 24’ × ‘GPBD 4’). The F_1_ plants were used as pollen parents to make the first backcross. In order to derive two more backcrosses (2nd and 3rd), selected BC_1_F_1_ and BC_2_F_1_ plants from each cross were used as pollen parents after foreground selection. Further, BC_2_F_1_ and BC_3_F_1_ plants were selfed to obtain segregating backcrossed F_2_ (BC_2_F_2_ and BC_3_F_2_). Such F_2_ generations were subjected to selection of homozygotes and disease screening (Fig. 1).

### Phenotyping for rust disease, yield and yield-related traits

Phenotyping of MABC lines for rust was done by creating artificial disease epiphytotics using the “spreader row technique” at Patancheru (India). Spreader rows of ‘TMV 2’ (the national susceptible check for both rust and LLS diseases in India) were planted on every tenth row inside the experimental plot and on border rows around the field to maintain effective inoculum load. The rust urediniospores were isolated by soaking and rubbing the infected leaves in water for 30 min and used for inoculation. After 45 days of sowing, the plants were uniformly inoculated with rust for a week in the evening, as detailed in Khedikar et al. (2010) and Sujay et al. (2012). Disease scoring for rust was done at 75 and 90 days after sowing (DAS) in different seasons by using a modified 9-point scale (Subbarao et al. 1990). In addition, stringent visual phenotypic criteria was used to select promising plants for important traits such as branching pattern, plant vigor, maturity duration, pod shape and size, seed weight and pod yield.

Additional disease screening and phenotyping for yield and yield-related traits was conducted in a replicated field experiment at ICRISAT, Patancheru, during the 2013 rainy season. Three separate evaluation trials with two replications were conducted in the rust disease nursery, each trial consisting of ILs, the donor and the respective recurrent parent genotype and check variety. ILs of ‘JL 24’ were evaluated in 5 × 5 alpha lattice design while ILs of ‘ICGV 91114’ and ‘TAG 24’ were evaluated in randomized block design (RBD) with 18 and 21 entries, respectively. Each entry was planted in four-row plots (4 m long, inter-row distance of 30 cm and intra-row distance of 10 cm). The experiment received 60 kg/ha P_2_O_5_ as basal dose and 400 kg/ha gypsum at peak flowering time (40 DAS). Seed was treated with Captan and Thiram fungicides in a 1:1 ratio @ 3 g/kg to protect against soilborne diseases. Sowing was done by hand and care was taken to ensure uniform planting at 5 cm depth. The crop was protected from weeds by the application of pre-emergence herbicide Pendimethalin (Stomp) at 1.0–1.5 kg/ha. Fungicidal Bavistin was sprayed at 1.0–1.5 L^−1^ to control LLS.

## Results

### Marker-assisted backcrossing (MABC)

Marker-assisted backcrossing with elite varieties including crossing, backcrossing and selfing was undertaken as mentioned in Fig. 1. The number of plants selected in each generation for marker screening and the number of positive plants (heterozygous in case of F_1_, BC_1_F_1_, BC_2_F_1_ and BC_3_F_1_ and homozygous in case of BC_2_F_2_ and BC_3_F_2_) are given in Table 2 and ESM Table 1.

**Table 2:** Summary of backcrossed and selfed plants sampled and positive plants identified in different generations

Generations	ICGV 91114		JL 24		TAG 24		Total plants	
	Plants screened	Plants positive	Plants screened	Plants positive	Plants screened	Plants positive	Plants screened	Plants positive
F_1_	36	32	37	30	32	25	105	87
BC_1_F_1_	94	23	46	14	46	16	186	53
BC_2_F_1_	71	13	55	21	68	22	194	56
BC_3_F_1_	115	35	35	8	31	11	181	54
BC_2_F_2_	75	31	25	3	214	53	314	87
BC_3_F_2_	216	40	112	30	37	4	365	74

As a first step, the rust resistance donor genotype ‘GPBD 4’ was used as the male parent and crossed individually with three susceptible elite varieties (‘ICGV 91114’, ‘JL 24’ and ‘TAG 24’) during 2008–2009 post-rainy season (December–April). From these crosses, harvested pods yielded 229 F_1_ seeds. Subsequently, 107 F_1_ seeds were planted during the 2009 rainy season (June–October). However, only 105 F_1_ plants were screened with the single marker IPAHM103, the then available marker from QTL analysis based on the partial genetic map. As a result, 87 F_1_ plants were found to be “true” hybrids carrying the target allele from ‘GPBD 4’ (Table 1). Thirty-two F_1_ plants from ‘ICGV 91114’ × ‘GPBD 4’, 30 plants from ‘JL 24’ × ‘GPBD 4’ and 25 plants from ‘TAG 24’ × ‘GPBD 4’ were used as pollen parents to make the first backcross (BC_1_) with the respective recurrent parents. From these crosses, 120 BC_1_F_1_ seeds from ‘ICGV 91114’ × ‘GPBD 4’, 63 from ‘JL 24’ × ‘GPBD 4’ and 52 from ‘TAG 24’ × ‘GPBD 4’ were harvested in October 2009.

A total of 235 BC_1_F_1_ seeds collected were planted in December 2009 in the 2009–2010 post-rainy season. One hundred and eighty-six BC_1_F_1_ plants were used for foreground selection with a total of four markers (IPAHM103, GM1536, GM2301 and GM2079). As a result, 53 BC_1_F_1_ plants (23 from ‘ICGV 91114’ × ‘GPBD 4’, 14 from ‘JL 24’ × ‘GPBD 4’ and 16 from ‘TAG 24’ × ‘GPBD 4’) were found heterozygous. Subsequently, all 53 positive plants were used to make the second backcross with the respective parents. A total of 211 BC_2_F_1_ seeds were harvested from these backcrosses in April 2010.

After sowing the 211 BC_2_F_1_ seeds in June 2010 (rainy season), 194 BC_2_F_1_ plants were raised and screened with all four SSR markers for foreground selection. Fifty-six plants, including 13 from ‘ICGV 91114’ × ‘GPBD 4’, 21 from ‘JL 24’ × ‘GPBD 4’ and 22 from ‘TAG 24’ × ‘GPBD 4’ were found heterozygotes with SSR markers. These plants were selected to make the third backcross (BC_3_) and 181 BC_3_F_1_ seeds were harvested. Similarly, all 194 BC_2_F_1_ plants were used for selfing and 339 BC_2_F_2_ pods (498 seeds) were harvested in October 2010.

In the next post-rainy season of 2010–2011, 181 BC_3_F_1_ and 498 BC_2_F_2_ seeds were planted in December 2010. Upon screening, 181 BC_3_F_1_ plants with all the four markers, 54 plants, including 35 from ‘ICGV 91114’ × ‘GPBD 4’, 8 from ‘JL 24’ × ‘GPBD 4’ and 11 from ‘TAG 24’ × ‘GPBD 4’ were identified as heterozygous and were selfed. From these 54 BC_3_F_1_ plants, 365 BC_3_F_2_ seeds were harvested in April 2011. At the same time, screening of 314 out of 498 BC_2_F_2_ plants led to the identification of 87 BC_2_F_2_ plants with homozygous resistant alleles for all the four SSR loci. Selfing of these plants provided BC_2_F_2:3_ seeds that were harvested at the end of the post-rainy season of 2011 in April.

During the 2011 rainy season, all the 365 BC_3_F_2_ seeds were sown. Foreground selection of these plants revealed that 74 BC_3_F_2_ plants (40 from ‘ICGV 91114’ × ‘GPBD 4’, 30 from ‘JL 24’ × ‘GPBD 4’ and 4 from ‘TAG 24’ × ‘GPBD 4’) possessed resistant alleles at all the four loci in homozygous state.

### Disease screening of MABC lines

In the 2011 rainy season, the 365 BC_3_F_2_ plants grown in the fields were also screened for disease resistance. All the 74 BC_3_F_2_ plants possessing homozygous alleles at all the four loci also showed resistant phenotype. The disease score for the homozygous plants ranged from 1.5 to 3.0. During the same season, 87 BC_2_F_2:3_ plant progenies were screened for rust disease, from which plants with high resistance and good agronomic features were selected. The 87 homozygous BC_2_F_2:3_ plant progenies showed good resistance ranging from 2.0 to 3.0 on a 1–9 scale at 90 DAS while recurrent parents recorded a disease score of 7.0. Seeds were harvested from 109 single plant selections (SPS) from 87 BC_2_F_2:3_ plant progenies.

In the 2011–2012 post-rainy season, 74 BC_3_F_2:3_ plant progenies and 109 SPS (BC_2_F_4_) were sown in rows. As a result, 83 BC_3_F_4_ and 117 BC_2_F_5_ seeds were harvested respectively in April 2012. The lines showed early maturity duration similar to the recurrent parents.

**Fig. 2:**
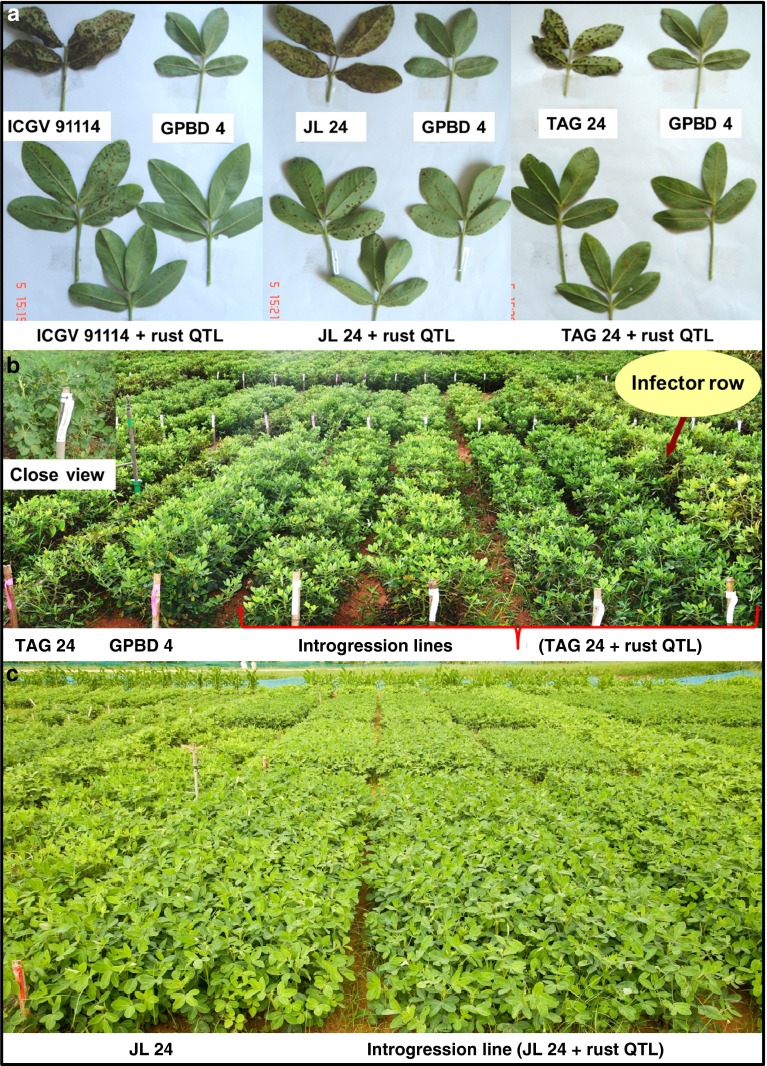
Disease reaction for rust resistance on parental genotypes and introgression lines (ILs) possessing rust QTL introgressed through marker-assisted backcrossing (MABC) in peanut. The *first panel* (**a**) showed disease reaction on susceptible and resistant parental genotypes; the *second panel* (**b**) showed disease reaction on leaves of parental and improved lines; and, field view of ILs possessing rust QTL through MABC programme. The *third panel* (**c**) showed replicated field evaluation of selected ILs of ‘JL 24’. The recurrent parent (‘JL 24’) and its ILs (‘JL 24’ + rust QTL) had similar phenotypic features

In the 2012 rainy season, 83 BC_3_F_4_ and 117 BC_2_F_5_ plant progenies were grown in two replications and were subjected to disease screening. In addition, stringent phenotypic selection criteria were imposed to select promising plants, comprising of desirable agronomic traits such as branching pattern, plant vigor, maturity duration, pod shape and size, seed weight and pod yield. Finally, a total of 81 improved ILs (19 BC_3_F_4_ and 62 BC_2_F_5_) with a disease score of 2.0 (on a scale of 1–9) were selected for further seed multiplication and yield assessment. All the ILs showed on par resistance to the donor parent genotype (‘GPBD 4’), i.e., disease score of 2.0 at 90 DAS. Their maturity duration and other yield parameters were similar to that of their respective recurrent parents. During the same screening season, all the three susceptible parents revealed a disease score of 5.0 at 90 DAS (Fig. 2). During the 2013 rainy season too, majority of the tested ILs showed a disease score of 2.0 or 2.5 at 90 DAS, a score similar to that of the donor parent genotype ‘GPBD 4’ while the recurrent parents had a much higher score at 90 DAS (6.5 in ‘ICGV 91114’, 7.0 in ‘JL 24’ and 6.0 in ‘TAG 24’) (Table 3). Similarly, the disease score for the ILs at 75 DAS was between 1.0 and 2.0, which is much lower than their respective recurrent parent genotypes.

**Table 3:** Rust resistance in ten best backcross introgression lines of ‘ICGV 91114’, ‘JL 24’ and ‘TAG 24’ during rainy 2012 and 2013 seasons

S no	Genetic background/introgression line	Rainy 2012		Rainy 2013		Average	
		Score @75 DAS	Score @90 DAS	Score @75 DAS	Score @90 DAS	Score @75 DAS	Score @90 DAS
‘ICGV 91114’ (Recurrent parent)		3.0	5.0	3.5	6.5	3.3	5.8
‘GPBD 4’ (Donor parent)		1.0	2.0	1.5	2.5	1.3	2.3
‘TMV 2’ (Susceptible check)		3.0	5.0	3.0	8.0	3.0	6.5
1	RBC2F5R12_13	2.0	2.0	2.0	2.0	2.0	2.0
2	RBC2F5R12_15	1.5	2.0	2.0	2.0	1.8	2.0
3	RBC2F5R12_16	2.0	2.0	1.0	2.0	1.5	2.0
4	RBC2F5R12_17	2.0	2.0	2.0	2.0	2.0	2.0
5	RBC2F5R12_18	1.5	2.0	2.0	2.0	1.8	2.0
6	RBC2F5R12_19	1.5	2.0	2.0	2.0	1.8	2.0
7	RBC2F5R12_23	2.0	2.0	2.0	2.0	2.0	2.0
8	RBC2F5R12_25	2.0	2.0	2.0	2.0	2.0	2.0
9	RBC2F5R12_29	1.0	2.0	2.0	2.0	1.5	2.0
10	RBC2F5R12_30	1.0	2.0	2.0	2.0	1.5	2.0
‘JL 24; (Recurrent parent)		3.0	5.0	4.0	7.0	3.5	6.0
‘GPBD 4’ (Donor parent)		1.0	2.0	1.5	2.5	1.3	2.3
‘TMV 2’ (Susceptible check)		3.0	5.0	3.0	8.0	3.0	6.5
11	RBC2F5R12_45	1.0	2.0	1.0	2.0	1.0	2.0
12	RBC2F5R12_46	1.0	2.0	1.5	2.0	1.3	2.0
13	RBC2F5R12_78	1.0	2.0	2.0	2.0	1.5	2.0
14	RBC2F5R12_87	1.5	2.0	2.0	2.0	1.8	2.0
15	RBC2F5R12_88	1.0	2.0	1.5	2.0	1.3	2.0
16	RBC2F5R12_97	1.0	2.0	2.0	2.0	1.5	2.0
17	RBC2F5R12_138	1.0	2.0	1.5	2.0	1.3	2.0
18	RBC2F5R12_139	1.0	2.0	2.0	2.0	1.5	2.0
19	RBC2F5R12_140	1.0	2.0	2.0	2.0	1.5	2.0
20	RBC2F5R12_143	1.0	2.0	1.5	2.0	1.3	2.0
‘TAG 24’ (Recurrent parent)		2.0	5.0	3.0	6.0	2.5	5.5
‘GPBD 4’ (Donor parent)		1.0	2.0	1.5	2.5	1.3	2.3
‘TMV 2’ (Susceptible check)		3.0	5.0	3.0	8.0	3.0	6.5
21	RBC2F5R12_103	1.0	2.0	2.0	2.0	1.5	2.0
22	RBC2F5R12_104	1.0	2.0	1.0	2.0	1.0	2.0
23	RBC2F5R12_107	1.0	2.0	2.0	2.0	1.5	2.0
24	RBC2F5R12_108	1.0	2.0	2.0	2.0	1.5	2.0
25	RBC2F5R12_114	1.0	2.0	2.0	2.0	1.5	2.0
26	RBC2F5R12_117	1.0	2.0	1.0	2.0	1.0	2.0
27	RBC2F5R12_118	1.0	2.0	1.0	2.0	1.0	2.0
28	RBC2F5R12_129	2.0	2.0	2.0	2.0	2.0	2.0
29	RBC2F5R12_130	1.0	2.0	2.0	2.0	1.5	2.0
30	RBC2F5R12_133	1.0	2.0	1.0	2.0	1.0	2.0

*DAS* days after sowing; *S no 1–10* ILs from the cross ‘ICGV 91114’ × ‘GPBD 4’; *S no 11–20* ILs from the cross ‘JL 24’ × ‘GPBD 4’; *S no 21–30* ILs from the cross ‘TAG 24’ × ‘GPBD 4’

### Yield assessment of ILs under disease infection

A replicated evaluation trial was conducted during the 2013 rainy season at ICRISAT-Patancheru to compare pod yield and yield-related traits of ILs (13 ILs of ‘ICGV 91114’, 21 ILs of ‘JL 24’ and 17 ILs of ‘TAG 24’) with their respective recurrent and donor parent genotypes. A good variation in pod and kernel yield, shelling percentage and 100-seed weight was observed among the ILs while a non-significant difference was seen for days to flowering. Twenty ILs with morphological features similar or very close to their respective recurrent parents were identified on the basis of growth habit, branching pattern, plant vigour, pod and seed feature. The 100-seed weight in ILs ranged from 31–39 g in the genetic background of ‘ICGV 91114’, 34–46 g in the genetic background of ‘JL 24’ while that of ‘TAG 24’ ranged from 31–39 g. Enhanced 100-seed weight was observed in several ILs (31 g in ‘ICGV 91114’, 32 g in ‘JL 24’ and 34 g in ‘TAG 24’) compared to their respective recurrent parent genotypes. More importantly, 8 of the 15 ILs of ‘ICGV 91114’ recorded 29–96 % higher pod yield (1,865–2,817 kg/ha) than the recurrent parent genotype ‘ICGV 91114’ (1,438 kg/ha). Ten of the 22 ILs of ‘JL 24’ recorded higher pod yield of 20–56 % (2,868–3,734 kg/ha) compared to ‘JL 24’ (2,400 kg/ha). Eight of the 17 ILs evaluated recorded 22–89 % higher pod yield (2,314–3,583 kg/ha) than the recurrent parent genotype ‘TAG 24’ (1,893 kg/ha). Yield parameters for one of the best ILs in the genetic background of each recurrent parent genotypes are given in Table 4. Several of the ILs showed a disease score on par with that of the donor parent genotype while displaying morphological features similar to that of the recurrent parent genotypes.

**Table 4:** Details of one of the best introgression line in each recurrent parent background of ‘ICGV 91114’, ‘JL 24’ and ‘TAG 24’

IL/recurrent parent	Days to flowering	Pod yield (kg/ha)	Pod yield gain (%)	Kernel yield (kg/ha)	Shelling percent (%)	100 seed weight (g)	Rust score @75 DAS	Rust score @90 DAS
RBC2F5R12_13 (IL of ‘ICGV 91114’)	31	1942	35.0	1417	73	38	2.0	2.0
‘ICGV 91114’	31	1438	–	1,078	75	31	3.5	6.5
RBC2F5R12_49 (IL of ‘JL 24’)	29	3,083	28.4	2,219	72	34	2.0	2.0
‘JL 24’	32	2,400	–	1,488	62	33	4.0	7.0
RBC2F5R12_104 (IL of ‘TAG 24’)	31	2,598	37.2	1,793	69	32	1.0	2.0
‘TAG 24’	31	1,893	–	1,306	69	34	3.0	6.0

### Tracking linkage drag in the carrier linkage group

**Fig. 3:**
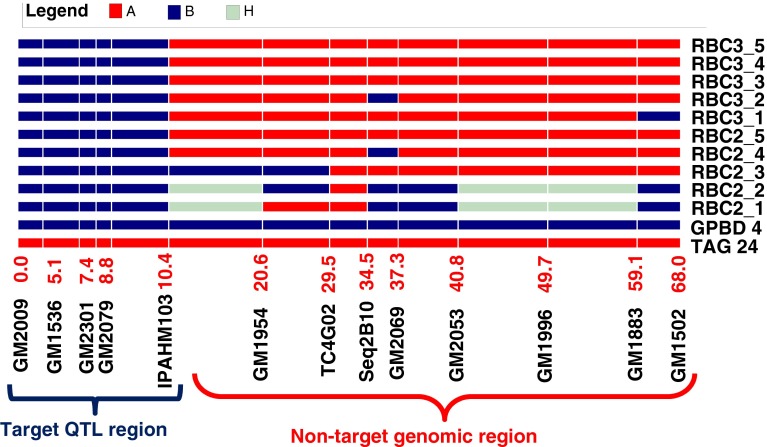
Monitoring genome introgression from the carrier linkage group among introgression lines (ILs). The first five samples are from second backcross (RBC2_1 to RBC2_5) while last five samples are from third backcross (RBC3_1 to RBC3_5) in the genetic background of ‘TAG 24’. RBC2F_5 among second backcross ILs showed same extent of higher maximum RP alleles in non-target genomic regions as shown by RBC3_3, RBC3_4 and RBC3_5 among third backcross derived lines

Of the selected 81 improved ILs, 43 representative ILs including 10 BC_2_F_2_ plants each from ‘ICGV 91114’ × ‘GPBD 4’, ‘JL 24’ × ‘GPBD 4’ and ‘TAG 24’ × ‘GPBD 4’, 10 BC_3_F_2_ plants from ‘ICGV 91114’ × ‘GPBD 4’ and 3 BC_3_F_2_ of ‘ICGV 91114’ × ‘GPBD 4’, with a disease score of 2.0, were genotyped with 13 SSR markers present on the carrier linkage group (AhXV). Of the 13 markers, 5 markers (GM2009, GM2301, GM1536, GM2079 and IPAHM103) have shown tight linkage for rust resistance (Sujay et al. 2012). Thus, only eight markers located on only one side of the QTL genomic region could provide an idea about the presence of donor segments in selected ILs. As expected, ILs derived from the third backcross possessed maximum alleles from the recurrent parent for eight markers compared to ILs derived from the second backcross. Despite this, lines with maximum recurrent parent genome (RPG) alleles were also observed and selected in second backcross progenies, but in very less frequency compared to third backcross-derived ILs where frequency was higher. For example, in the genetic background of ‘TAG 24’, BC2_5 plant (now in BC_2_F_5_ generation as RBC2F5R12_114) from BC_2_F_2_ had maximum RPG alleles, BC3_4 (now in BC_3_F_5_ generation as RBC3F4R12_61) and BC3_5 plants (now in BC_3_F_5_ generation as RBC3F4R12_97) from BC_3_F_2_ had maximum RPG allele (Fig. 3).

## Discussion

Leaf rust together with late leaf spot make a devastating combination in peanut and adversely affect yield and fodder quality. Millions of smallholder farmers choose peanut for both food and fodder (dual-purpose) which remains the mainstay of their livelihood. Marker-assisted backcrossing has been proven to be a quick way to improve one or two traits in existing preferred cultivars in several crops (Varshney et al. 2010). Foliar fungal diseases in peanut damage crop yield as well as spoil the quality of leaves which smallholder farmers use as fodder for their animals. Therefore, making popular varieties resistant to foliar diseases will sustain not only the productivity of the cultivars but also the livelihoods of the farmers. We report the successful improvement of three popular elite peanut varieties because of their special features such as high yield, early duration, drought tolerance, and medium bold kernels, among others.

An initial QTL analysis using partial genetic map for ‘TAG 24’ × ‘GPBD 4’ (Khedikar et al. 2010) led to the identification of only one linked marker, IPAHM103, with moderate phenotyping variance (55.20 %). However, the resistant allele for this marker was validated in a range of germplasm set including another mapping population (‘TG 26’ × ‘GPBD 4’). Hence this marker was immediately deployed for initiating MABC in three varieties with the objective of introgression of the QTL genomic region controlling rust resistance. Since IPAHM103 was dominant in nature, efforts were on to saturate genetic maps for both populations (‘TAG 24’ × ‘GPBD 4’ and ‘TG 26’ × ‘GPBD 4’), with a special interest in saturating the QTL genomic region controlling rust resistance. A detailed analysis provided four additional SSR markers with increased phenotypic variance (82.20 %) mapped in the same QTL genomic region. Three (GM1536, GM2301 and GM2079) of the four identified markers showed good amplification and thus were confirmed for polymorphism among parental genotypes (‘ICGV 91114’, ‘TAG 24’, ‘JL 24’, and ‘GPBD 4’). As a result, three newly identified co-dominant markers (GM1536, GM2301 and GM2079) along with IPAHM103 were deployed in screening the BC_1_F_1_ generation onwards in all backcross progenies of the three varieties.

After undertaking second and third backcrosses, the backcrossed ILs were selfed in all the three crosses. As expected, while screening segregating ILs, co-dominant markers proved helpful in selecting plants containing homozygous allele from the donor parent. As a result, 200 ILs from all crosses were selected. Screening them for rust resistance led to the identification of 81 lines possessing a disease score of 2.0 (on a 1–9 scale), which is on par with the disease score observed for the donor, ‘GPBD 4’.

While screening ILs, a difference in disease severity was observed between the 2011 rainy season (where susceptible parents scored 7.0) and 2012 rainy season (where susceptible parents scored 5.0). Replicated disease screening of selected ILs under field conditions during the 2013 rainy season showed that majority of the ILs had a lower disease score similar to that of the donor parent (‘GPBD 4’) while the recurrent parents recorded a much higher score during the same crop duration. Due to heavy incidence of disease on the susceptible recurrent parents, the higher parts of the plants including the stems turned black. Consequently, they had to be harvested a week earlier along with the infector row (‘TMV 2’). This indicates that these markers can avoid confusion while selecting plant progenies under fluctuating environments. It was interesting to note that majority of the backcross-derived lines were found to be early maturing, similar to their respective recurrent parents. This was in contrast to conventional rust resistance breeding where a high level of resistance to rust was often found associated with longer crop duration.

Replicated yield assessment under disease infection showed increase in pod yield by up to 96 % higher than ‘ICGV 91114’, 56 % more than ‘JL 24’ and 89 % higher than ‘TAG 24’. The higher pod yield in ILs compared to that in the recurrent parent may be partly attributed to the protection offered by resistance QTL genomic region against the fungal pathogen. Most importantly, the differences were not significant for days to flowering, indicating that ILs had similar maturity as their respective recurrent parent genotypes.

While MABC commonly employs background selection, this study did not do so as the donor genotype (‘GPBD 4’) is an elite variety. Therefore, even after 2–3 backcrosses with the recurrent varieties, some segments from ‘GPBD 4’ come in the ILs, linkage drag is not anticipated. Nevertheless, screening of improved ILs with 13 markers on the carrier linkage group showed 11 lines that possess only the targeted QTL region of the donor ‘GPBD 4’.

Our earlier study also observed that the targeted QTL region also has QTLs that contribute to LLS resistance (67.98 % PV) (Sujay et al. 2012). Therefore, it is anticipated that the ILs developed in this study will display some level of resistance against LLS as well. An interesting feature of the ILs is the high level of resistance they display not only until the stage of full maturity but also at harvesting stage. Hence, in addition to having a positive impact on yield, the ILs will also enhance fodder quality.

Apart from peanut, rust has also been adversely affecting wheat, maize, soybean, etc. which belong to an elite group of crops with huge genomic resources to conduct genetic studies and employ molecular breeding. Although several studies have reported QTLs for resistance to rust in wheat (Da-Silva et al. 2012; Zhang et al. 2013), maize (Collins et al. 1999; Kerns et al. 1999; Brown et al. 2001), sorghum (Tao et al. 1998; Peng et al. 1999), barley (van Berloo et al. 2001), soybean (Hyten et al. 2007, 2009), sunflower (Qi et al. 2012; Bulos et al. 2013), pea (Rai et al. 2011), flax (Bo et al. 2002), and oat (Zhu and Kaeppler 2003; Portyanko et al. 2005; McCartney et al. 2011), molecular breeding for rust resistance has been reported in only some cases. For instance, simple marker-assisted selection has been done to check the efficiency of linked markers in improving rust resistance in sunflower (Lawson et al. 1998) and wheat (Bariana et al. 2007; Mago et al. 2009). The MABC approach was recently used to improve rust resistance in wheat (Randhawa et al. 2009) and sunflower (Bulos et al. 2013).

This study is the first of its kind for developing superior lines for rust resistance not only in peanut but in any legume crop. It is the third molecular breeding study for developing superior lines in peanut. The other studies reported the development of ‘NemaTAM’, a line with resistance to root knot nematode (Simpson et al. 2003) and the development of ‘Tifguard High O/L’ with a high ratio of oleic:linoleic acid (O/L) (Chu et al. 2011). Though these two studies developed superior lines for root knot nematode (Simpson et al. 2003) and high oleate trait (Chu et al. 2011), molecular markers used in these studies were not identified through genetic mapping, as has been the case in the present study.

## Summary

The study improved rust resistance in three leading Indian peanut varieties—‘ICGV 91114’, ‘JL 24’ and ‘TAG 24’. For instance, ‘ICGV 91114’ is grown on 800,000 ha in drought-prone Anantapur district (India) which has world’s largest peanut-growing area in a single district (Birthal et al. 2011). Its rising popularity is evident from the increased demand for breeder seed in the All India Coordinated Project on Groundnut (3rd highest indented variety after ‘Kadiri 6’ and ‘TMV 2’) which went up from 11.56 tons during 2009–2010 to 20.0 tons during 2012–2013. On the other hand, ‘JL 24’ and ‘TAG 24’ stood at seventh and fifth place respectively in terms of indented varieties of India during 2012–2013 (http://www.nrcg.res.in/index.php?option=com_content&view=article&id=7&Itemid=8). However, all these varieties are susceptible to rust disease (score of 5.0–6.0 at 90 DAS). This study has produced ILs from these varieties with a rust disease score of <2.0 with significant increase in pod yield. Therefore, after multi-location field trials, the ILs have potential to be released as improved varieties in India that will eventually lead to greater yield and income to resource-poor farmers of the semi-arid tropics (SAT). They may also be released in other parts of the world with similar agroclimatic conditions.

This study also revealed that rust resistance can be combined with early maturity. It is common for advanced breeding lines to have a disease score of 2.0, but they are all late maturing. The SAT needs short-duration varieties with enhanced disease resistance. Furthermore, successful introgression of resistance with four markers underlines the value of the markers in undertaking MABC to improve elite but rust-susceptible varieties. The study also demonstrates the accelerated development of rust-resistant varieties with the ultimate aim of peanut improvement.

## Electronic supplementary material

Below is the link to the electronic supplementary material.

Supplementary material 1 (XLS 32 kb)
